# Early markers of cardiovascular injury in childhood leukaemia survivors treated with anthracycline chemotherapy

**DOI:** 10.1186/s40959-019-0047-4

**Published:** 2019-08-14

**Authors:** Treya M. Long, Channa E. Marsh, Lawrence G. Dembo, Philip Watson, Karen E. Wallman, Thomas S. Walwyn, Catherine S. Choong, Louise H. Naylor

**Affiliations:** 1 School of Human Sciences: Exercise and Sport Science, The University of Western Australia, 35 Stirling Highway, Perth, WA 6009 Australia; 2 Envision Medical Imaging, 178-190 Cambridge St, Wembley, WA 6014 Australia; 3 Advanced Heart Failure Unit and Cardiac Transplant Service of Western Australia, Fiona Stanley Hospital, 11 Robin Warren Dr, Murdoch, WA 6150 Australia; 4 School of Medicine: Paediatrics, The University of Western Australia, 35 Stirling Highway, Perth, WA 6009 Australia; 5 Department of Haematology and Oncology, Perth Children’s Hospital, 15 Hospital Ave, Nedlands, WA 6009 Australia; 6 Department of Endocrinology, Perth Children’s Hospital, 15 Hospital Ave, Nedlands, WA 6009 Australia

**Keywords:** Cardiac magnetic resonance imaging, Childhood leukaemia, Anthracyclines, Late cardiotoxicity, Long-term survival

## Abstract

**Background:**

Cardiovascular disease (CVD) is the leading non-malignant cause of death in childhood cancer survivors. Heightened risk of CVD is often attributable to treatment with anthracycline chemotherapy. Anthracycline-mediated cardiac injury may lie latent for years following cessation of treatment and is therefore often not detected until disease is advanced and aggressive therapy is required. Symptomatic CVD may be preceded by subclinical cardiac and vascular dysfunction. This study aimed to determine whether such dysfunction could be detected in healthy, anthracycline-treated survivors of childhood leukaemia.

**Methods:**

Cardiac magnetic resonance imaging (cMRI) with late gadolinium enhancement and endothelial function were used to characterise pre-clinical stages of CVD. Twenty-two long-term (>5 years survival; age 21 ± 3 years) childhood leukaemia survivors were assessed. All survivors were asymptomatic and had normal resting echocardiography. To exclude potential confounding effects of radiotherapy, no survivors had received this treatment. Twenty-two similarly aged (25 ± 3 years) gender-matched controls were recruited for comparison.

**Results:**

Left ventricular ejection fraction was lower in the survivors (55.0 ± 4.6%) compared to the controls (59.4 ± 6.2%; *p* = 0.010). Further, five survivors (23%) had clinically reduced (<50%) left ventricular ejection fraction. Normalised left ventricular end systolic volume was augmented in survivors (40.0 ± 9.1 mL·m^2^ vs. 34.5 ± 7.5 mL·m^2^; *p* = 0.038). Cardiac MRI did not show any late gadolinium enhancement. High resolution, ultrasound-derived flow mediated dilation was impaired in survivors (6.7 ± 2.1% vs. 8.60 ± 1.91%, *p* = 0.005).

**Conclusions:**

We detected subclinical changes in cardiovascular structure and function indicative of early disease in anthracycline-treated childhood leukaemia survivors with normal echocardiography. Early detection and characterisation of underlying disease allows for timely intervention and improved outcomes in this at-risk population.

## Background

The current five-year survival rate for childhood cancer in developed countries is approaching 85% [1–3]. Unfortunately, these gains come at a cost – the long-term impact of malignancy and its treatment is substantial, with 62% of survivors developing a chronic health condition within 17 years of diagnosis [4]. Cardiovascular disease (CVD) presents a significant burden, with childhood cancer survivors 3.4 times more likely to suffer cardiac-related death by their late 30’s compared to the general population [5]. Anthracycline chemotherapy has been identified as an independent risk-factor for CVD, with studies showing that almost half of exposed survivors will develop injury to the heart [6–9]. Unfortunately, there is often a long latency period between anthracycline exposure and clinically evident disease where survivors are free of signs and symptoms; this makes early prevention problematic [10]. Further, there are currently very few effective ways of detecting subclinical cardiac dysfunction in survivors [10]. This emphasises the need to find effective follow-up screening measures that will allow for early detection of underlying abnormalities before they become too advanced and difficult to treat.

Cardiac magnetic resonance imaging (cMRI) has high spatial resolution and superb contrast resolution; it also has very low inter and intra observer variability and is the gold-standard for assessment of cardiac morphology [11–14]. Contrast agents such as gadolinium can be used in conjunction with cMRI to detect changes in myocardial extracellular volume that may reflect inflammation or fibrosis. As a healthy myocardium is densely packed with viable myocytes that do not permit entrance of gadolinium into the cell, the extracellular space is small and thus there is little gadolinium enhancement of a normal myocardium [11, 15]. However, based on the volume and distribution of gadolinium, pathologies resulting in increased extracellular space (e.g. fibrosis, inflammation, infiltration) may be detected and defined with cMRI using late gadolinium enhancement [11, 15]. The few studies that have utilised cMRI in cancer survivor populations show promise regarding its ability to detect structural and functional abnormalities relating to various cancer therapies [12, 13, 16–19]. However, those studies that have included late gadolinium enhancement have reported inconsistent results regarding its efficacy at detecting myocardial pathologies characteristic of latent injury [17–19]. Unfortunately, cancer diagnosis and treatment type are not always regulated in studies and it can be difficult to make conclusions about cardiovascular toxicity with regards to specific populations and/or therapies. Additionally, it is rare to see cMRI studies that utilise matched control groups. Leukaemia is the most common childhood cancer, with its survivors representing one of the largest cohorts at risk of, or currently experiencing, the various complications of late anthracycline toxicity [8, 20–23]. Despite this, it has not yet been determined whether cMRI with late gadolinium enhancement is able to detect signs of underlying anthracycline injury in this survivorship group. Further, anthracycline toxicity has not been characterised in this cohort based on comparisons with a healthy, matched control group.

Endothelial dysfunction has been independently established as an early event in the progression of CVD [24, 25]. Moreover, there is evidence to suggest that the endothelial cells are more sensitive to reactive oxygen species produced during anthracycline treatment than the cardiomyocytes [26, 27]. It is unknown whether there is an association between vascular health and early cardiac dysfunction in those leukaemia survivors who have been treated with anthracyclines.

Early characterisation of subclinical disease is important for maximising the time available to test interventions commonly used for other types of heart disease, until an appropriate course of treatment is identified. In the present study, we used cMRI with late gadolinium enhancement to assess cardiac structure, function and tissue composition in a cohort of adolescent and young adult (AYA) survivors of childhood acute lymphoblastic leukaemia (ALL) and acute myeloid leukaemia (AML) who were treated with anthracyclines but no radiotherapy. Secondary to this, we also performed an assessment of endothelial function. These results will allow us to make a novel analysis of cardiovascular health that will potentially allow for improved outcomes due to earlier characterisation and more timely and targeted intervention.

## Methods

### Participants

**Fig. 1:**
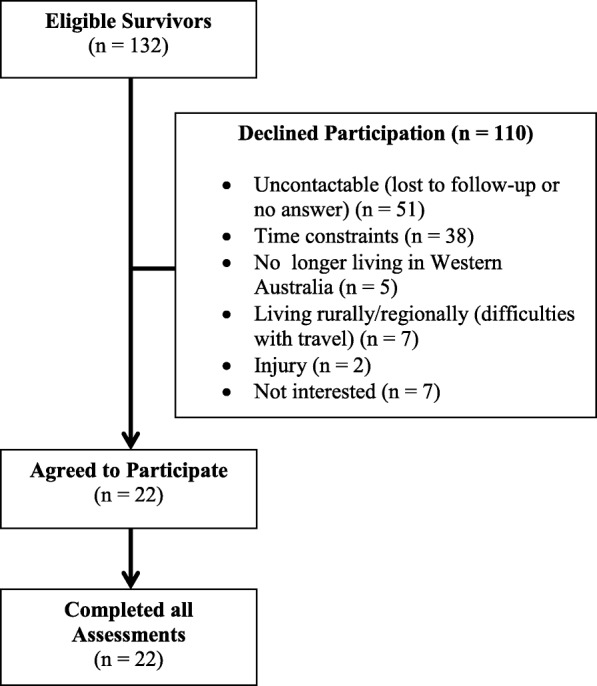
Study recruitment flow diagram

The Princess Margaret Hospital for Children (Western Australia) oncology database was searched in order to identify long-term (≥5 years) AYA (15–30 years) survivors of childhood ALL and AML. Those who had received radiotherapy as part of their treatment protocol were excluded, as were survivors with any abnormalities of cardiac structure or function (ie. presentation of ventricular dilation, systolic and/or diastolic dysfunction, wall thickness or wall motion abnormalities, pericardial effusions or endocarditis) in their most recent echocardiograms (all within 2 years of the study). Survivors with a history of acute and/or early-onset cardiac toxicity were not excluded if current echocardiograms were deemed normal by a cardiologist. Eligible survivors were either approached by study clinicians during long-term follow-up clinics or were called by study clinicians if appointments had not been attended or were not scheduled within the recruitment time-frame. If survivors consented to receive further information about the study, the primary researcher emailed them official information sheets and then performed a phone call to discuss the study, answer any questions and, if necessary, arrange written consent. Of 132 eligible survivors, 22 (11 males, 11 females; age range, 15–25 years) consented to participation and completed all necessary assessments. The primary reasons for non-participation were loss to follow-up and a lack of time (Fig. 1). Twenty-two similarly aged (range, 19–29 years), gender matched healthy individuals were also recruited from the community to act as a control group for the study.

Ethical approval for this study was granted by the Princess Margaret Hospital Human Research Ethics Committee (approval number, 2016108EP) and the University of Western Australia Human Research Ethics Committee (reference number, RA/4/1/9090). All participants (or parents/guardians for those under 18 years) were required to provide informed, written consent prior to participation.

### Experimental design

Participants were asked to fast for a minimum of 4 h and to avoid exercise for 24 h prior to their initial testing session. During this session, measurement of anthropometry, resting heart rate (HR) and resting blood pressure (BP) occurred, followed by evaluation of endothelial function using the flow mediated dilation (FMD) technique. Cardiac MRI’s were performed after the survivors were cleared for gadolinium infusion via assessment of renal function. Control participants did not receive gadolinium.

### Anthropometry

Height, body mass, waist circumference and hip circumference were assessed according to protocols detailed by The American College of Sports Medicine [28]. Height was measured using a wall-mounted stadiometer (Seca 216 Measuring Pole, Birmingham, UK), while body mass was measured using an electronic scale (Sauter Model EB60, FSE Scientific, New South Wales, Australia). A girth tape was used to measure waist circumference and hip circumference. Body mass index (BMI) was calculated from measurements of height and body mass using formula published by The World Health Organisation [29]. Waist-to-hip ratio was calculated by dividing waist circumference by hip circumference.

### Vascular function

Participants lay supine for 20 min before having their resting HR and BP assessed using an automated sphygmomanometer (Dinamap CARESCAPE V100, GE Healthcare, General Electric Company, Buckinghamshire, UK). Endothelial function was then assessed using the FMD technique described by Thijssen et al. [25]. Recordings of the brachial artery were captured using non-invasive, high-resolution ultrasound (uSmart 3300 NexGen, Terason, Burlington, Massachusetts, USA). A 1 min preliminary recording of brachial artery diameter and blood flow velocity was taken following the placement of a pneumatic cuff on the forearm, distal to the olecranon process. This cuff was then inflated to 220 mmHg for 5 min following baseline imaging, with recordings of diameter and blood flow re-commencing at 4 min 30 s and continuing for 3 min after cuff deflation. Details regarding analysis and analysis software are presented elsewhere [25]. Baseline, peak and delta diameter were measured in mm, while FMD was calculated as the percentage of diameter change. Time to peak diameter was recorded in seconds and converted to minutes for graphical representation.

### Cardiac magnetic resonance imaging

Left and right ventricular (LV and RV, respectively) morphology were assessed using cMRI (1.5 T MAGNETOM Aera, Siemens, Erlangen, Germany). Participants were scanned in a supine position, with the examination taking approximately 30 min. To improve image quality and spatial resolution over the standard body coil, a posterior phased array spinal coil and an anterior flexible phased array body surface coil were used whilst imaging. Standard cardiac imaging planes were obtained using multi-plane breath hold True FISP localisers. Breath hold times ranged between 5 and 20 s, depending on the participants resting HR. In order to assess functional parameters, breath hold True FISP, electrocardiogram-gated cine images consisting of 10–15 individual slices (6 mm slices/4 mm gap, FoV 320–350 mm, TR = 37.68, TE = 1.29, flip angle 70–80 degrees, resolution 256 × 166, BW 930) were acquired. Cine images of the LV and RV were acquired in the short axis plane, perpendicular to the ventricular septum. Long axis cine images were acquired in the axial plane (from level of pulmonary valve to diaphragm) to better visualise the RV free wall. Four chamber cine views and LV outflow tract cine images (6 mm slices, FoV 300–330 mm, TR = 38.28, TE = 1.32, flip angle 70–80 degrees, resolution 224 × 224, BW 930) were also taken to assess valve function, myocardial wall motion and blood flow through the heart.

Late gadolinium enhancement imaging was acquired in the leukaemia survivor group to detect the possible presence of treatment-induced myocardial tissue pathologies. Gadoteric acid (0.15 mmol/kg infused at 3 mL/second, Dotarem, Guerbet, Roissy, France) was administered via an intravenous cubital fossa injection. After a 5 min delay, a short axis mid-ventricular inversion time Scout (TrueFISP sequence) was acquired to determine the inversion time for the nulling of normal myocardial signal. Within the next 5–10 min, the late gadolinium enhancement images were obtained using both standard inversion recovery and Phase Sensitive Inversion Recovery sequences (inversion time range, 250–350 milliseconds). A slice thickness of 8 mm was used in the short axis, 2-chamber view (vertical long axis through the left atrium and ventricle), 3-chamber view (LV outflow tract) and 4-chamber view (horizontal long axis).

### Data analyses

Cardiac MRI’s were analysed by the primary researcher (TML) using Siemens specialised cardiac analysis software (syngo.via VB20A_HF04). In the initial phase of analysis, the primary researcher was unable to be blinded to participant status as scans were taken clinically and had been saved under each participant’s name. However, an experienced radiographer (PW) who was blinded to participant status repeated all analysis to reduce bias and ensure consistency of figures. Additionally, a blinded cardiologist (LGD) confirmed the final data set.

Reported measures were automatically calculated by the analysis software following segmentation of appropriate anatomical regions and structures. Systemic and pulmonary outflow tracts were auto-segmented in individual slices on cine images and then manually adjusted where necessary using a nudging tool. Short axis cines were visually inspected to identify end diastolic and end systolic frames for ventricular analysis. The end diastolic frame was selected as the one with the largest ventricular cavity/greatest blood pool (typically the first frame of the series), while the end systolic frame had the smallest ventricular cavity/lowest blood pool. An accompanying cine volume graph confirmed selection of end diastolic and end systolic frames. Marking of the mitral valve on two and four chamber cine images for the LV was automated but manually adjustable if tracking was inconsistent over frames. Manual placement was required for markers of the tricuspid valve annular plane in RV analysis. The basal frame was taken as the first complete slice below the level of the mitral and tricuspid valves. Endocardial and epicardial borders of the LV were automatically contoured on each individual frame and then manually adjusted using nudging and drawing tools. The RV endocardium was manually traced on each short axis frame. Papillary muscles were included in the blood pool in both systole and diastole. Data was internally validated by confirming that aortic flow, pulmonary flow, LV stroke volume and RV stroke volume were all within 5% of each other. Any deviations resulted in re-analysis and, if required, review by a cardiologist.

All gadolinium scans were visually inspected by a cardiologist (LGD) who determined whether any myocardial morphological abnormalities or intra-cardiac shunts were present.

### Statistical analyses

Statistical analysis was performed using IBM SPSS version 20.0 (IBM Australia Ltd., New South Wales, Australia). Differences between cancer survivors and controls were identified using independent samples t-tests, with significance set at *p* ≤ 0.05.

## Results

### Participant characteristics

Twenty-two (11 male, 11 female) leukaemia survivors participated in the study and completed all assessments. The average age of the leukaemia survivors was 21 ± 3 years (range 15–25 years). Mean age at diagnosis was 6 ± 4 years, resulting in an average time since diagnosis of 14 ± 4 years. The average time since final treatment was 13 ± 4 years. All survivors entered the study with completely normal echocardiograms. Half of the survivors (*n* = 11) had no history of cardiac toxicity, while the other half had a history of acute and/or early-onset cardiac toxicity that recovered in the years following treatment. Additional treatment and diagnostic information is presented in Table 1. Cumulative anthracycline dosages were calculated based on guidelines provided by the Children’s Oncology Group [30]. The control participants were matched based on gender (ie. we recruited 11 male and 11 female controls). The controls had a mean age of 25 ± 3 years (range 19–29 years).

**Table 1:** Characteristics of the leukaemia survivors

	Survivor (*n* = 22)	
	No.	%
Underlying Diagnosis		
Leukaemia	22	100
ALL	15	68
Standard Risk	13	
Intermediate Risk	0	
High Risk	2	
AML	7	32
Standard Risk	6	
Intermediate Risk	1	
High Risk	0	
Treatment		
Chemotherapy	20	91
Chemotherapy & HSCT^a^	2	9
Treatment Details		
Anthracycline Chemotherapy	22	100
Cumulative Anthracycline Dosage		
< 100 mg·m^2^	9	41
100–249 mg·m^2^	6	27
250–399 mg·m^2^	7	32

*ALL* Acute lymphoblastic leukaemia, *AML* Acute myeloid leukaemia, *HSCT* Haematopoietic stem cell transplant

^a^HSCT was used in the treatment of two AML patients. Total body irradiation was not part of the conditioning schedule

The controls had a mean systolic BP of 118 ± 11 mmHg, a diastolic BP of 65 ± 9 mmHg, a mean arterial pressure of 85 ± 9 mmHg and a resting HR of 63 ± 9 bpm. The leukaemia survivors had similar mean systolic and diastolic BPs (113 ± 11 mmHg, *p* = 0.089 and 62 ± 6 mmHg, *p* = 0.254) and a comparable mean arterial pressure of 82 ± 7 mmHg (*p* = 0.249). However, the survivors had a higher resting HR than the controls (71 ± 9 bpm, *p* = 0.010).

### Anthropometry

The leukaemia survivors had an average height of 173.0 ± 7.7 cm, a body mass of 76.15 ± 19.05 kg and a BMI of 25.2 ± 5.1. Height (173.8 ± 9.1 cm, *p* = 0.796), body mass (70.07 ± 14.0 kg, *p* = 0.287) and BMI (22.9 ± 2.7, *p* = 0.109) were not significantly different in the control group. With regards to circumferences, the survivors and the controls had similar waist (84.1 ± 12.6 cm vs. 77.8 ± 10.0 cm, respectively; *p* = 0.107) and hip measurements (98.7 ± 12.1 vs. 92.9 ± 7.1 cm, respectively; *p* = 0.093). Similarly, waist-to-hip ratio was not different between groups (survivors, 0.85 ± 0.07; controls, 0.84 ± 0.07; *p* = 0.554).

### Vascular function

**Fig. 2:**
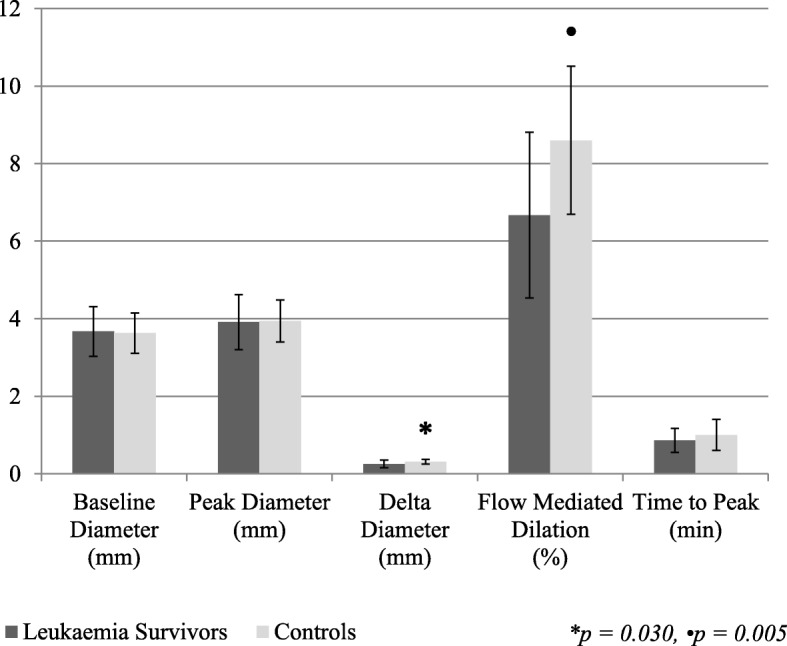
Endothelial function data for leukaemia survivors and controls. Error bars represent standard deviation for each measure

The survivors had a lower FMD than the controls (6.7 ± 2.1% vs. 8.6 ± 1.9%, *p* = 0.005), as can be seen in Fig. 2. Delta diameter was also reduced in the survivor cohort (0.25 ± 0.10 mm vs. 0.31 ± 0.06 mm, *p* = 0.030). Despite this, there were no differences between survivor and control groups for baseline diameter (survivors, 3.67 ± 0.64 mm; controls, 3.63 ± 0.52 mm; *p* = 0.843), peak diameter (survivors, 3.91 ± 0.71 mm; controls, 3.94 ± 0.54 mm; *p* = 0.915), or time to peak (survivors, 0.86 ± 0.31 min; controls, 0.99 ± 0.40 min; *p* = 0.234).

### Cardiac magnetic resonance imaging

**Fig. 3:**
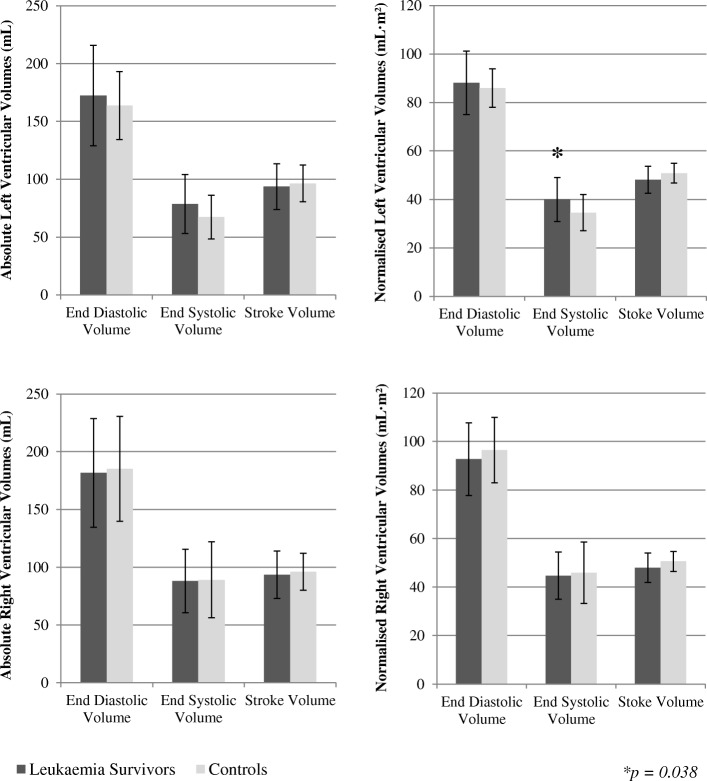
Left and right ventricular absolute and normalised blood volumes for leukaemia survivors and controls. Error bars represent standard deviation for each measure

Left ventricular ejection fraction (EF) was significantly reduced in the leukaemia survivors compared to the controls (55.0 ± 4.6% vs. 59.4 ± 6.2%; *p* = 0.010). Five survivors (23%; 1 ALL with a cumulative anthracycline dosage of 150 mg·m^2^ and no history of cardiotoxicity. Four AMLs with a cumulative anthracycline dosage ≥340 mg·m^2^, two of which had mildly dilated LVs acutely during treatment and two with no history of cardiotoxicity) had clinically reduced LVEF (defined as <50% [31–33]), while all the controls were normal. Despite this, there were no differences between groups in RVEF (leukaemia survivors, 52.1 ± 3.5%; controls, 53.2 ± 7.1%; *p* = 0.516). Figure 3 shows that there were no differences between survivors and controls for measures of absolute and normalised LV end diastolic volume (*p* = 0.443 and p = 0.516, respectively) and absolute or normalised stroke volume (*p* = 0.622 and *p* = 0.079, respectively). There was no difference between leukaemia survivors and controls for absolute LV end systolic volume (*p* = 0.106), although once normalised for body surface area LV end systolic volume was higher in the survivors (*p* = 0.038). There were no significant differences between groups for any absolute or normalised measures of the RV (Fig. 3). Measures of LV myocardial mass did not differ between leukaemia survivors and controls, nor did LV and RV cardiac output and cardiac index (Table 2).

**Table 2:** Cardiac MRI parameters for leukaemia survivors and controls

	Survivors (*n* = 22)	Controls (*n* = 22)	*P* value
LV Cardiac Output (L·min^−1^)	6.8 ± 1.4	6.2 ± 1.1	0.117
LV Cardiac Index (L·min^− 1^·m^2^)	3.5 ± 0.5	3.2 ± 0.4	0.077
RV Cardiac Output (L·min^− 1^)	6.8 ± 1.5	6.1 ± 1.1	0.123
RV Cardiac Index (L·min^− 1^·m^2^)	3.5 ± 0.5	3.2 ± 0.4	0.088
LV Myocardial Mass at ED (g)	110.1 ± 33.4	104.9 ± 32.6	0.600
LV Myocardial Mass at ED (g·m^2^)	55.7 ± 10.9	54.3 ± 10.7	0.670
Average LV Myocardial Mass (g)	108.4 ± 31.9	103.6 ± 30.3	0.611
Average LV Myocardial Mass (g·m^2^)	54.6 ± 10.4	53.8 ± 9.9	0.784

*MRI* Magnetic resonance imaging, *LV* Left ventricular, *RV* Right ventricular, *ED* End diastole

Gadolinium scans did not unmask any fibrosis or other myocardial changes.

## Discussion

In the present study, cMRI detected subclinical changes in cardiac structure and function suggestive of potential future disease in a cohort of healthy, asymptomatic childhood leukaemia survivors treated with anthracyclines. This study has also highlighted the presence of vascular endothelial dysfunction that may accelerate progression of anthracycline injury, as well as predispose to concomitant CVD in this population.

Importantly, none of the survivors included in the current study had received radiotherapy and all were free from cardiac symptoms and had normal resting echocardiography. Despite this, cMRI uncovered significantly reduced LVEF in the leukaemia survivors compared to the healthy controls. Furthermore, 23% of these survivors were detected to have clinically reduced (<50% [31–33]) LVEF. These findings highlight a decline in cardiac function following anthracycline chemotherapy which is predominantly silent for an average of 13 years following the end of leukaemia treatment. Additionally, our findings have demonstrated that even those leukaemia survivors treated with relatively low cumulative anthracycline dosages <250 mg·m^2^ are at risk of such deterioration and disease. Similar findings have been presented by Armstrong et al. [16], who reported that 27% of their childhood cancer survivors who were previously undiagnosed with cardiac dysfunction and treated with cumulative anthracycline doses <150 mg·m^2^ had LVEF two standard deviations below normative ranges. The present study both validates and strengthens these findings through the use of a healthy control group and provides a novel analysis of cardiac function in an isolated cohort of childhood leukaemia survivors. Interestingly, the survivors included in the Armstrong et al. [16] study had a significantly longer time since diagnosis (27.74 years, range 18–38 years) than our cohort, indicating earlier detection of disease in our childhood leukaemia survivor population is possible. This timely detection allows for earlier intervention, which will help to maximise treatment outcomes.

A reduction in LVEF can be attributable to a decrease in end diastolic volume or an increase in end systolic volume [34]. In the current study, we observed augmented normalised LV end systolic volume in the leukaemia survivors when compared to the control participants. In keeping with our findings, Ylänen et al. [17] observed increased LV end systolic volume in anthracycline-treated childhood cancer survivors upon assessment with cMRI. However, the cohort utilised by Ylänen et al. [17] included survivors who had also received adjuvant radiotherapy. Therefore, our results provide a unique picture of cardiovascular toxicity development in AYA survivors of acute childhood leukaemia who only received anthracyclines.

In the anthracycline-exposed individual, increased ventricular volumes and cardiac dysfunction typically result from underlying myocardial injury caused by cancer treatment – for example, myocardial fibrosis, myofibrillar lysis, infiltrative disorder and myocellular dysfunction [34–36]. However, in the present study there was no late gadolinium enhancement on any of the survivor cMRIs and no differences in myocardial mass between groups. Similar to our study, Ylänen et al. [17] also failed to see changes in late gadolinium enhancement scans used on a mixed group of childhood cancer survivors with a median follow-up of 7.8 years. Additionally, Ylänen et al. [17] did not find differences in myocardial mass between their survivor cohort and referenced normative data, despite observing abnormalities in EF and ventricular volumes. Together, our findings suggest that there is minimal fibrosis involved in the early remodelling process (ie. larger LV volumes and lower LVEF) raising the question as to whether there is any value in including late gadolinium enhancement into cMRI follow-up scans whilst cancer survivors are within the first two decades of survival. However, it is important to note that our lack of findings may also be due to imaging procedures used and/or the inability of current measures to detect subclinical myocardial changes in this cohort. Interestingly, despite the similarities in findings, the survivor group used in the study by Ylänen et al. [17] had received radiotherapy. This indicates that early progression of late anthracycline toxicity is similar between childhood cancer survivors irrespective of treatment with other therapies.

Anthracycline chemotherapy induces oxidative stress that is toxic to the vascular endothelium [37, 38]. Endothelial injury reduces the ability of the vessels to vasodilate and greatly increases susceptibility to accelerated atherosclerosis; hence, FMD is a strong predictor of cardiovascular events [24, 25]. In the current study we observed a significantly reduced FMD in our cohort of asymptomatic childhood ALL and AML survivors. Notably, this occurred despite similarities between survivor and control groups in resting BP, body mass, BMI and waist circumference. While endothelial dysfunction has previously been demonstrated in survivors of childhood ALL [39, 40] we are among the first to perform this measure in an isolated leukaemia survivor population that did not receive radiotherapy. Additionally, the inclusion of a matched control group strengthens the validity of our FMD findings. Impaired endothelial function may lead to poor peripheral vasodilation which can, in turn, increase systemic vascular resistance, resulting in an increase in afterload [38]. Increased afterload can lead to a concomitant increase in LV end systolic volume, which was a prime observation in the current study. Our combined observations imply a relationship between endothelial dysfunction and the development of late cardiac toxicity in this cohort. This finding will need to be corroborated by larger studies.

There is currently no consensus on the best management or treatment options for cardiovascular complications relating to anthracycline chemotherapy [41–44]. Unfortunately, once abnormalities are clinically observable and/or symptomatic there is often limited time left to trial different treatment strategies, resulting in varied results that are often short-lived [41–44]. For example, mortality from restrictive cardiomyopathy – a common late presentation of anthracycline cardiotoxicity – can be as high as 50% within 2 years of diagnosis [35, 45]. This emphasises the importance of studies such as ours, which help to characterise the earliest changes in cardiovascular structure and function that may indicate future disease and, therefore, allow for timely intervention. This is enforced by a study in adult cancer survivors, which showed better outcomes with earlier initiation of treatment for patients with diagnosed anthracycline cardiomyopathy [46].

It may be considered that a limitation of this study is the small sample size. However, when using cMRI the sample size needed to detect a 3% change in EF, a 10 mL change in end diastolic volume and end systolic volue, and a 10 g change in myocardial mass is 81–97% smaller than the sample size that is needed to detect the same differences when using standard echocardiography (range: 9–15 patients using cMRI vs. 53–273 patients using echocardiography) [47]. Further, this study was considerably strengthened by the use of a healthy, matched control group. As there is variability between machines, technicians, analysis procedures and software, normative data is not the most accurate basis for comparisons. This can be seen when comparing our healthy control group to the normative data used by Armstrong et al. [16], with male controls in our study presenting with a LVEF (56.3%) almost two standard deviations below the mean LVEF reported for healthy males aged 20–39 years (64.3%) [48]. While we have reported on some of the more common CVD risk-factors here (eg. resting BP, BMI and waist circumference), we were unable to collect data on lifestyle factors (eg. smoking history and physical activity status) that may have contributed to the development of endothelial dysfunction in our leukaemia survivors. However, we are confident that the anthracycline treatment was the primary variable influencing our results. In a previous study we reported normal endothelial function in AYA survivors of childhood brain cancer who presented with significantly reduced cardiorespiratory fitness [49]. This indicates that endothelial abnormalities caused by such risk-factors are slow to develop and, therefore, may not have had any effect on our leukaemia survivor endothelial function results.

## Conclusions

The present study has demonstrated that cMRI is successful in unmasking early subclinical changes in cardiac structure and function indicative of future disease in asymptomatic childhood leukaemia survivors exposed solely to anthracycline chemotherapy. While gadolinium enhancement did not appear to add any additional value to scans in this stage of survival, cMRI alone allowed for nearly one-quarter of the survivors to be referred for cardiology review, possibly saving future costs of having to treat advanced diseases and allowing for better outcomes due to prompt initiation of treatment. In addition to this, the current study has demonstrated the importance of also assessing endothelial health in this cohort as a means of investigating risk of exacerbated and/or concomitant CVD.

## Data Availability

The datasets used and/or analysed during the current study are available from the corresponding author on reasonable request.

## Abbreviations

ALL: Acute lymphoblastic leukaemia
AML: Acute myeloid leukaemia
AYA: Adolescent and young adult
BP: Blood pressure
cMRI: Cardiac magnetic resonance imaging
CVD: Cardiovascular disease
EF: Ejection fraction
FMD: Flow mediated dilation
HR: Heart rate
LV: Left ventricle
RV: Right ventricle
